# Tasked with a Challenging Objective: Why Do Neutrophils Fail to Battle *Pseudomonas aeruginosa* Biofilms

**DOI:** 10.3390/pathogens8040283

**Published:** 2019-12-04

**Authors:** Jennifer Geddes-McAlister, Abirami Kugadas, Mihaela Gadjeva

**Affiliations:** 1 Molecular and Cellular Biology Department, University of Guelph, Guelph, ON N1G 2W1, Canada; jgeddesm@uoguelph.ca; 2 Division of Infectious Diseases, Department of Medicine, Brigham and Women’s Hospital, Harvard Medical School, Boston, MA 02115, USA; abiramikugadas10@gmail.com

**Keywords:** neutrophils, biofilms, therapies, enzymes

## Abstract

Multidrug-resistant (MDR) bacterial infections are a leading cause of mortality, affecting approximately 250,000 people in Canada and over 2 million people in the United States, annually. The lack of efficacy of antibiotic-based treatments is often caused by inability of the drug to penetrate bacterial biofilms in sufficient concentrations, posing a major therapeutic challenge. Here, we review the most recent information about the architecture of *Pseudomonas aeruginosa* biofilms in vivo and describe how advances in imaging and mass spectroscopy analysis bring about novel therapeutic options and challenge existing dogmas.

*Pseudomonas aeruginosa* is a Gram-negative, opportunistic pathogen with sophisticated mechanisms for evasion of immune responses. Low nutritional requirements and high tolerance to stress makes *P. aeruginosa* extremely dangerous. It causes nosocomial pneumonias associated with artificial ventilation, chronic obstructive pulmonary disease, chronic lung infections in Cystic Fibrosis (CF) patients, surface infections in wounds and in burned patients, and keratitis [1,2]. During infection, *P. aeruginosa* utilizes many secreted and cell-associated virulence factors, including toxins (e.g., ExoS, ExoT, and ExoU) [3], which promote bacterial evasion of the host immune responses, and enzymes (e.g. protease IV), which degrade complement proteins, immunoglobulins, and fibrinogen [4]. Here, we focus on key characteristics of *P. aeruginosa* biofilms that mediate host evasion. 

## 1. Chasing *Pseudomonas aeruginosa* through Their Own Small Molecules

Interbacterial communication governs population survival under different environmental conditions. Bacteria release small molecules to regulate the growth patterns of the population and alter responses of the host. These systems are termed quorum sensing (QS). *P. aeruginosa* has sophisticated QS systems, comprising the Lux homologues LasRI and RhlRI. LasRI and RhlRI function in a hierarchical manner in controlling the gene expression. LasI and RhlI are responsible for the synthesis of *N*-(3-oxododecanoyl) homoserine lactone (3-oxo-C12-HSL) and *N-*butanoylhomoserine lactone (C4-HSL), respectively, while the LasR and RhlR function as receptors for 3-oxo-C12-HSL and C4-HSL and subsequently activate gene expression of QS target genes [5]. In addition to these two systems, a third signaling molecule, “pseudomonas quinolone signal” (PQS), which is intertwined between the *las* and *rhl* systems exists [6]. These signaling systems control expression of a multitude of virulence factors (e.g., elastase, rhamnolipids, and pyocyanin) and biofilm dynamics [7]. Consistently, *P. aeruginosa* mutant strains that do not express QS signals have reduced virulence compared with wild-type strains [8]. A recent study shows that one of the key QS molecules, N-3-oxo-dodecanoyl-l-homoserine lactone (C12) induces apoptosis of neutrophils, another mechanism by which *Pseudomonas aeruginosa* evades the host immune response [9]. Hence, capturing QS molecules in the microenvironment can prevent bacterial communication and interfere or reduce biofilm formation as well as enable neutrophils to phagocytose and eliminate *Pseudomonas* cells [10,11]. These treatments are not bactericidal in nature, but they can augment the host’s immunity. Enzymes that inactivate QS signals are called quorum quenching enzymes (QQE), while chemicals that disrupt QS pathways and reduce the expression of QS-controlled genes are called quorum sensing inhibitors (QSI) [12]. The presence of multiple QS systems in individual bacterial species poses a challenge in QS interference strategy since multiple QS systems might have different induction dynamics during the bacterial growth. Combination therapy targeting quorum quenching enzyme and quorum sensing inhibitor completely blocked *P. aeruginosa las* and *rhl* QS systems in vitro [13]. Future studies should interrogate the efficacy of this approach in vivo. QSI such as itaconimides cooperate with tobramycin to reduce *P. aeruginosa* biofilms. The synergy is rather spectacular as the QSI target the base of the biofilm while antibiotics target the peripheral bacterial growth, but detailed mechanistic understanding of the therapeutic effect is still missing [14].

A downstream event consequent to QS is synthesis of a secondary messenger, 3’,5’-cyclic diguanylic acid (cyclic(c)-di-GMP). The c-di-GMP molecule is composed of two guanosine-5’-triphosphate (GTP) residues and it is synthetized by 3’5’-diguanyl cyclase protein. Conversely, the degradation of c-di-GMP depends on phosphodiesterase activity. The dynamic intracellular levels of 3’5’-diguanyl cyclase and phosphodiesterase control c-di-GMP concentrations and how bacterial cells adapt to the environment [15,16]. The c-di-GMP has multiple functions. It works by (i) allosteric modifications of enzymes, (ii) interaction with transcription factors, and (iii) interactions with srRNA (riboswitch) [17,18]. The c-di-GMP is a positive regulator for polysaccharide biosynthesis including alginate and Pel. c-di-GMP works both locally and globally. An extensive overview of c-di-GMP biology is not a subject of this review, but there are excellent reviews describing in detail its function [19]. c-di-GMP is emerging as an attractive target for clinical intervention. A screen for inhibitors of virulence among a library of fungal metabolites identified a five-ring ketone containing a molecule termed terrein, which reduced virulence factors secretion and biofilm production. Terrein inhibited both QS and cyclic-di-GMP signaling and caused a decrease in virulence in an infection model study [20]. Cumulatively, these reports demonstrate that targeting signaling molecules associated with *P. aeruginosa* growth and virulence represent promising directions for new therapeutics.

## 2. A Structure Designed to Protect

Biofilms are defined as high-density bacterial clusters, frequently attached to surfaces and encased in an extracellular polymeric matrix [21]. *P. aeruginosa* biofilm formation includes a complex set of well-coordinated events. In vitro studies in flow cells have shown that bacteria, after initial attachment to surfaces (e.g., glass, plastics, or biological surfaces), serve as focal points for microcolony formation. Once settled in the developing microcolonies, bacteria become encapsulated in protective extracellular polymeric substance (EPS) matrix, consisting of polysaccharides, proteins, and extracellular DNA (eDNA) [22,23,24]. Upon maturation, the microcolonies undergo seeding dispersal, resulting in the formation of a multitude of bacterial biofilms. Each of the bacterial biofilms poses internal cavities, which contain motile cells that, upon rupture of the biofilm exterior, are freed from the biofilm [25].

Bjamsholt et al. studied biofilms in explanted lungs from CF patients via fluorescent imaging and in situ hybridization [26]. Biofilms subjected to extensive antibiotic treatment were found in the conductive zone of the lungs of CF patients, while autopsies from patients lacking aggressive antibiotic treatment showed biofilms in the conductive and respiratory zones [27]. They also found that biofilms consisted of small aggregates, usually containing less than a thousand bacteria, and they were not adherent to the epithelium. These findings illustrate that patients with chronic infections carry a multitude of biofilm structures.

We imaged *P. aeruginosa* biofilms in a mouse model of ocular keratitis [28,29]. This model mimics an acute infection of the eye, which rapidly progresses and can result in vision loss. Biofilm formation dynamics were monitored and rapid biofilm assembly was observed within the first 48 h of infection. Consistently, cryo-electron microscopy imaging also showed that *P. aeruginosa* formed bacterial microclusters on ocular surfaces [24]. Taken together, these findings challenged the traditional view that only chronic infections were readily associated with biofilms. 

**Figure 1:**
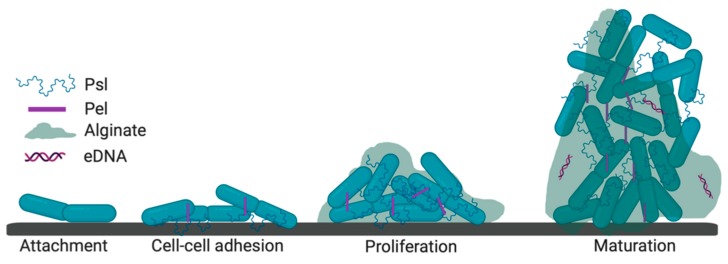
Proposed model for biofilm architecture. *P. aeruginosa* biofilms are surrounded by Psl (polysaccharide synthesis locus) in the periphery, Pel (pellicle) is centrally located, while eDNA can be found in the biofilm core and in the periphery, where it surrounds the biofilms participating in the formation of the “death zone”. Figure generated with Biorender.com.

As described above, biofilms represent bacterial communities within a protective EPS matrix, which surrounds live bacteria. In *P. aeruginosa,* EPS consists of polysaccharides: polysaccharide synthesis locus (Psl), pellicle (Pel), and alginate (Figure 1). Psl is a polymer of α-D-mannose, D-glucose, and L-rhamnose and is present in a cell-associated form or as a component of the EPS matrix [30]. Psl protects cells against phagocytes and oxidative stress [31]. The Psl-rich biofilms carry ecotin, a Psl-binding protein with serine protease inhibitory functions, which likely inhibits complement convertases, hence significantly inhibiting complement mediated opsonophagocytosis [32]. Psl also mediates cell attachment to abiotic and biotic surfaces and keeps bacteria trapped within the matrix [33]. Cell anchoring can occur via associations between Psl and the surface protein CdrA, which is required for cell–cell aggregation. 

**Figure 2:**
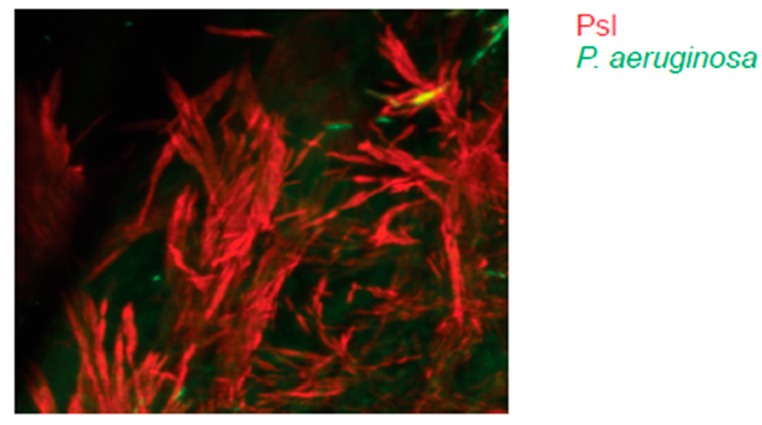
Psl fibers emanating from *P. aeruginosa* clinical isolate 6294 generated biofilms. C57BL6/N mouse was infected with 5 × 10^5^ cfu/eye *P. aeruginosa* 6294 expressing green fluorescent protein GFP. The intravital microscopy was carried out at 24h after the infectious challenge. Bacterial biofilm was stained with fluorescently labeled anti-Psl mAb Cam003 (red), which was applied topically 15 min before imaging, eyes were washed with phosphate buffered saline (PBS), mice were sedated with ketamine–xylasine anesthesia and imaged. Imaging across the cornea revealed Psl (red) fibers. Images are recorded at 40×.

Psl undergoes spatial rearrangement during microcolony formation and is relocated to the periphery of the biofilm (i.e., a Psl-free cavity) that is subsequently used in the aforementioned seeding dispersal of motile cells. Psl can form fiber-like structures, which have been observed in vitro [23] and may form similar structures in vivo. Indeed, we observed Psl fibers in vivo emanating from the body of the biofilms (Figure 2). These structures could have dual function—trapping of antibiotics [34] or other active compounds and/or, secondly, aiding bacterial motility and dispersion [35]. Consistently, the PAO1-derived strain with a mutation that inactivates Psl production was significantly impaired in the formation of biofilm [29]. Cumulatively, these data convincingly document the importance of Psl in biofilm formation.

Since the majority of keratitis- and CF-associated *P. aeruginosa* strains express Psl, it appears that this polysaccharide is a good target for drug development [36]. The prophylactic and therapeutic outcomes of the anti-Psl antibodies have been extensively studied [37]. It has a significant prophylactic potential [29], but despite the excellent in vitro opsonophagocytic activities of the antibodies raised against Psl, the therapeutic potential is limited [28]. Given the recently-reported benefits of the bispecific anti-Psl and anti-PcrV antibody in multiple animal infection models [38,39,40], it is likely that targeting of multiple virulence mechanisms is needed to confer protection. 

Another component of the EPS is Pel, a glucose-rich extracellular matrix polysaccharide, initially described for its role in pellicle formation (thin membranes) in an air–liquid interface [41]. Unlike Psl, Pel is not mediating surface attachment; however, similar to Psl, it binds eDNA, and, hence, can support early biofilm development [42]. Pel, in contrast to Psl, reduces elasticity and crosslinking of the biofilm matrix [42]. Consequently, levels of Pel are thought to reduce during biofilm maturation, thereby supporting dispersal. Consistently, Pel deficiency results in increased biofilm erosion [43]. To date, there is no imaging data to show Pel location and dynamics during biofilm growth in vivo. It also remains to be evaluated whether Pel is a good drug target.

Alginate is a negatively charged polymer consisting of β-D-mannuronic acid and α-L-guluronic acid, with roles in bacterial evasion of complement and preventing phagocytosis. Unlike Psl and Pel, alginate is not required for microcolony formation, but supports matrix integrity [44,45]. Alginate overproduction is called mucoid conversion and is a dominant event in CF patients associated with chronic infections of the lungs. Therefore, alginate may serve as a target for vaccine development in CF [46]. To date, human anti-alginate antibody has been developed and has shown therapeutic efficacy in mice during ocular or lung infections [47,48,49]. Unlike in CF, alginate does not appear to be dominant in the keratitis isolates and its absence does not negate biofilm formation [28]. These findings illustrate that *P. aeruginosa* uses distinct polysaccharides for niche adaptation and therefore, such site-specific polysaccharides should be targeted therapeutically.

Along with polysaccharides, proteins like type IV pili, cup fimbriae, CdrA adhesin, LecB lectin, and Fap amyloid compose the biofilm matrix [50]. Overexpression of *cdrA* and *cdrB*, the CdrA exporter, promote bacterial autoaggregation [51]. LecB binds bacterial polysaccharides, strengthening the biofilm [52,53]. Double mutant strains that lack both *cdrA* and *lecB* expression form a monolayer, but largely fail to retain Psl [53]. Reconstituting *crdA* expression was not sufficient to restore Psl biofilm architecture in vitro, suggesting that expression of *CrdA* or *LecB* alone were not enough under certain circumstances to recover Psl architecture [53]. It has been proposed that the two proteins (CrdA and LecB) may differ in their stability under unique environments or that there is still an unidentified protein maintaining the Psl architecture, illustrating the need for a thorough proteome-focused analysis of biofilms. 

Investigation of the biofilm-associated components demonstrate that overexpression of *fap* thickens biofilms. Whereas, CupA, CupB, CupC, and CupD fimbriae are involved in bacterial attachment and biofilm formation [54,55]. Lastly, Type IV pili form complexes with eDNA, facilitating biofilm establishment [56]. Cumulatively, these reports show a significant proteinaceous component to the biofilm formation. It still remains unclear how redundant the functions of these proteins are in supporting the biofilm architecture in niche-specific fashion, whether there are any niche requirements for their expression, or if these proteins present therapeutic targets. 

eDNA is a significant component of *P. aeruginosa* biofilms [23,57]. It has long been debated whether DNA in biofilms is of bacterial or host origin. In vitro-based flow chamber studies have shown that bacteria shed DNA to facilitate microcolony formation. Psl and Pel bind eDNA to strengthen biofilm structures, providing evidence that biofilm DNA is of bacterial origin [34]. Conversely, the presence of host-derived DNA may be associated with the ability of neutrophils to release neutrophil extracellular traps (NETs). During *P. aeruginosa* infection, release of NETs is induced; however, *P. aeruginosa* are capable of resisting NET-mediated killing [58,59,60,61,62]. Mechanistically, a multitude of factors contribute to NET release. For example, *P. aeruginosa* virulence factors such as Type 3 Secretion System (T3SS), an increase in release of LasB elastase and LasA protease, quorum sensing mechanisms, and bacterial motility stimulate NETosis [29,63].

The long-lasting debate of the importance of NETs during *P. aeruginosa* infection was put to rest by examining biofilm formation in mice that cannot produce NETs. Here, PAD4-deficient mice whose neutrophils cannot release NETs demonstrated lower levels of ocular inflammation and less biofilm formation [29]. However, *P. aeruginosa* disseminated to the brain tissues in these mice, which indicates that free-living cells were predominant in the absence of NETs, illustrating that host-derived NETs supported bacterial compartmentalization [29]. Cumulatively, these reports showed that NETs delayed bacterial dissemination. 

## 3. Can Enzyme-based Therapies Help?

*P. aeruginosa* biofilms are protected from neutrophil infiltration [28,29]. PMNs approach, but cannot penetrate. Unable to phagocytose, neutrophils release NETs, thereby building a “death zone”, which surrounds bacterial biofilms. These data suggest that intervention strategies to disrupt biofilm could improve neutrophil function by promoting access. These treatments are likely to be adjunctive to antibiotics since EPS components tend to capture and reduce the therapeutic activity of the antibiotic class such as aminoglycosides [57].

The classic example for an enzyme-based therapy that targets bacterial biofilms is the use of Pulmozyme (recombinant human DNase; dornase alpha). It is an FDA-approved drug for treatment of CF. Pulmozyme solubilizes CF patient sputum, improving lung function. Further, DNase treatment enhances antibiotic penetrance and efficacy due to alterations of biofilm structures [64]. Interestingly, we, and others, observed that small-molecular-weight DNA fragments less than 200 bp long had significant bactericidal activities [65]. Similarly, synthetic short dsDNA fragments were also bactericidal [65]. While not all tested CF isolates showed sensitivity to short dsDNA, the majority of the bacterial isolates did, allowing the intriguing conclusion that an aspect of Pulmozyme activity is the generation of short bactericidal eDNA fragments. Cumulatively, these results illustrate different mechanisms by which rhDNase limits pathogenesis.

Immature biofilms are more sensitive to DNase treatment than mature biofilms, posing the question of what alternatives exist for biofilm solubilization. An interesting new approach to dissolve biofilms came from the group of Dr. Howell (The Hospital for Sick Children, Toronto, CA), who proposed the use of exopolysaccharide glycoside hydrolases to target biofilms [66,67]. The Psl and Pel biosynthetic operons encode two enzymes: a putative periplasmic glucoside hydrolase family 39, termed PslG, and a PelA, a multifunctional protein with two catalytic domains, a glycoside hydrolase domain and a CE4-deacetylase domain. Fragments of these genes were recombinantly expressed, and the purified proteins successfully dissolved *P. aeruginosa* biofilms generated by CF clinical isolates. Further, both enzymes improved antibiotic efficacy. The enzymes had minimal cytotoxicity, which, when combined with the anti-Psl and anti-Pel specificities, make them promising novel drug options. Additionally, improved recovery from a wound infection was reported upon Psl glucoside hydrolase treatment, which correlated with greater complement deposition and improved neutrophil phagocytosis [67].

Another recent study demonstrated that α-mannosidase reduced in vitro biofilms [68]. We confirmed and expanded on these findings by providing evidence that neutrophil-derived α-mannosidases (Man2b1 and Man2c1) activity was needed for optimal bactericidal function against *P. aeruginosa* [28]. These findings were supported by total proteome profiling of bone marrow-derived neutrophils, which defined a difference in α-mannosidase (Man2b1 and Man2c1) production during infection [69]. We also showed that topical application of α -mannosidase decreases corneal bacterial burden in vivo [28]. In humans, mutations that impair lysosomal α -mannosidase (Man2b1) activity cause development of α-mannosidase, a condition that is rare, autosomal recessive, and multisystemic. This progressive lysosomal storage disorder results in facial and skeletal abnormalities, motor impairment, hearing impairment, intellectual disability, immune deficiency, and recurrent infections [70]. Importantly, the recombinant human α -mannosidase showed promise in clinical trials for enzyme replacement therapy [71]. The recombinant α -mannosidase was well tolerated; the frequency of infusion-related reactions and the development of allo-antibodies were low compared with other enzyme replacement therapies [71]. These findings are exciting as they suggest the use of recombinant human α -mannosidase as a treatment of biofilm-based infections.

## 4. Antimicrobial Peptides to the Rescue

A plethora of human antimicrobial peptides with anti-biofilm activities have been described, including defensins, fragments derived from thrombospondin, lacritin, and ApoB proteins [72,73,74,75]. With the exception of lacritin-derived peptides, the bactericidal activities of the thrombospondin-derived peptides or ApoB are within 1–20 µM ranges, making them less attractive therapeutics. However, the peptides showed significantly improved efficacy when combined with antibiotics. For example, cryptic host defense peptides derived from ApoB improved the bactericidal activity of colistin by 50% [76]. In contrast to these findings, LL-37, an antimicrobial peptide derived from cathelicidin, showed significant toxicity [77], restricting the potential use of the peptide to topical applications. Another interesting example is the random copolymer glatiramer acetate (GA), which, while is not a naturally occurring peptide, is similar to LL-37 and is antibacterial against keratitis and CF *P. aeruginosa* isolates [73]. Given that GA is the first effective medical treatment of multiple sclerosis (MS), the repurposing of this drug seems easily feasible. 

## 5. Implications and Future Directions

Detailed understanding of the kinetics and structure of *P. aeruginosa* biofilms can offer disease-associated markers that enable diagnosis of disease states and monitoring of disease progression. Combined therapies based on appropriate antibiotic treatment paired with biofilm-targeted strategies will improve antibiotic efficacies and control MDR development. Lastly, detailed characterization of the biochemical components of the biofilms, including protein and polysaccharide components, may point to novel vaccine targets.
